# Tryptophan metabolism: a critical bridge linking gut microbiota to depression

**DOI:** 10.3389/fmicb.2026.1936353

**Published:** 2026-09-17

**Authors:** Haoyuan Tan, Yuxin Pang, Qiaoyu Ji, Zhongxian Li, Junquan Liang, Ronghui Xian, Peng Zhou, Yaxin Zhang

**Affiliations:** The Seventh Clinical Medical College, Guangzhou University of Chinese Medicine, Shenzhen, Guangdong, China

**Keywords:** depression, gut microbiota, gut-brain axis, kynurenine pathway, tryptophan metabolism

## Abstract

Depression is a major global public health challenge with limitations in current therapies. Emerging evidence highlights that gut microbiota (GM) critically regulates the gut-brain axis via tryptophan (Trp) metabolism. Trp is metabolized through host-directed serotonin (5-HT) and kynurenine (KYN) pathways, as well as microbiota-specific indole derivative pathways. In depression, gut dysbiosis shifts Trp metabolism toward the KYN pathway, leading to accumulation of neurotoxic metabolites such as quinolinic acid and reduced 5-HT synthesis. Microbiota-derived indoles, functioning as key aryl hydrocarbon receptor (AhR) ligands, regulate blood-brain barrier integrity, neuroinflammation, and hypothalamic-pituitary-adrenal (HPA) axis function through AhR activation, thereby exerting neuroprotective or pro-inflammatory effects depending on the specific ligand and context. Interventions targeting the Trp-microbiota axis include dietary modifications, probiotics, antibiotics, traditional Chinese medicine, fecal microbiota transplantation, and metabolite supplementation. However, clinical translation is hindered by population heterogeneity, strain specificity, and insufficient dynamic monitoring. Future research should integrate multi-omics and neuroimaging approaches to improve patient stratification and advance Trp metabolism-based precision psychiatry, while emerging computational tools may further support biomarker integration and treatment prediction.

## 1 Introduction

Depression is a highly disabling public health issue. According to statistics from the World Health Organization (WHO), approximately 350 million people worldwide suffer from this condition; its high prevalence and widespread social impact have made it a considerable societal burden. According to the Global Burden of Disease (GBD) 2021 database, by 2021 the number of people with depression had exceeded 350 million globally, and the incidence, prevalence, and disability-adjusted life years (DALYs) of depression all showed an upward trend between 1990 and 2021 (GBD 2021 Diseases and Injuries Collaborators, 2024). Its primary clinical features include depressed mood, loss of interest, or fatigue, which severely affect patients’ quality of life and social functioning (Xu et al., 2026; Xu et al., 2026). Currently, first-line pharmacotherapy represented by selective serotonin reuptake inhibitors (SSRIs) is widely used in clinical practice (Jannini et al., 2022); however, their inconsistent efficacy, delayed onset of action, and potential adverse effects such as sexual dysfunction and sleep disturbances significantly limit therapeutic benefits (Golder et al., 2023). Approximately 30-50% of patients show a poor response to existing antidepressants. Faced with this challenge, exploring new mechanisms underlying the pathogenesis of depression and identifying safer and more effective therapeutic targets have become key research priorities in neuropsychiatry (Li J. et al., 2023).

The pathogenesis of depression is extremely complex, involving multiple factors such as genetics, endocrinology, and immune inflammation. In recent years, the proposal of the brain-gut axis mechanism has provided an entirely new perspective for understanding the pathophysiological processes of depression (Liu et al., 2023). As a key regulator in the bidirectional brain-gut communication system, the GM can influence central nervous system (CNS) function through its metabolic products. Among these, tryptophan (Trp) metabolism serves as the crucial biochemical bridge linking the GM to CNS function. Trp, an essential amino acid for humans, is metabolized primarily via two pathways: first, through the tryptophan hydroxylase (TPH) pathway to produce 5-hydroxytryptamine (5-HT, serotonin), which directly influences emotional regulation and neuroplasticity; second, via the indoleamine 2,3-dioxygenase (IDO1/IDO2) pathway into the kynurenine (KYN) pathway, generating a series of neuroactive metabolites (Gao et al., 2020). Patients with depression exhibit significant alterations in GM composition, characterized by a reduction in short-chain fatty acid (SCFA)-producing bacterial genera and an enrichment of pro-inflammatory bacteria. This dysbiosis can cause Trp metabolism to shift toward the KYN pathway, promoting the accumulation of neurotoxic metabolites such as quinolinic acid (QUIN), whilst simultaneously reducing the bioavailability of 5-HT, ultimately contributing to the onset and maintenance of depressive symptoms (Kennedy et al., 2017). More importantly, in states of neuroinflammation, pro-inflammatory cytokines can activate IDO, further exacerbating the shift of Trp metabolism toward the KYN pathway, thereby creating a vicious cycle of “microbiota dysbiosis-immune activation-neuroinflammation,” whilst also affecting the function of the hypothalamic-pituitary-adrenal (HPA) axis and gut homeostasis (Nayak et al., 2026). As a key link between the GM and the CNS, research into the regulatory mechanisms of Trp metabolism not only helps elucidate the pathological basis of depression but also provides a theoretical foundation for intervention strategies based on the gut microbiome. Accordingly, this paper systematically reviews the mechanisms by which Trp metabolism derived from the GM contributes to the pathogenesis of depression, with a view to providing new insights and targets for the diagnosis and treatment of depression.

## 2 Literature search strategy

This narrative review was based on a comprehensive literature search conducted in PubMed, Web of Science, and Scopus. Relevant studies published from January 2006 to July 2026 were identified using combinations of the following keywords: “tryptophan metabolism,” “kynurenine,” “serotonin,” “indole,” “gut microbiota,” “gut-brain axis,” “depression,” “major depressive disorder,” “aryl hydrocarbon receptor,” “fecal microbiota transplantation,” and “probiotics.” Reference lists of relevant reviews and original research articles were also screened to identify additional studies.

Priority was given to peer-reviewed original studies, clinical trials, meta-analyses, and high quality reviews directly addressing the relationships among GM, Trp metabolism, and depression. Studies focusing exclusively on unrelated neurological or metabolic disorders were excluded unless they provided mechanistic evidence relevant to Trp-mediated gut-brain communication. Because this article is intended as a narrative rather than a systematic review, no formal risk-of-bias assessment or meta-analysis was performed. The final literature set was selected on the basis of relevance to the major themes discussed in this review, including the kynurenine, serotonin, and microbial indole pathways, gut microbial dysbiosis, neuroimmune regulation, and microbiota-targeted interventions.

## 3 Clinical and experimental evidence linking gut microbiota dysbiosis to depression

A large number of observational clinical studies provide literature support for reduced GM diversity and altered abundance of specific bacterial genera in patients with depression. Lin et al., through 16S rRNA sequencing analysis of 106 antidepressant-naïve patients with depression and 151 healthy controls, found that the α-diversity of the GM in patients was significantly lower than that in the control group, and there were marked differences in β-diversity; patients exhibited a significant reduction in the relative abundance of the genera *Dialister* and *Lactococcus* (Lin et al., 2025), indicating that the GM of depressed patients undergoes adverse changes in both overall abundance and genus level composition, and that the reduction in beneficial bacterial genera may be closely associated with the onset and progression of depression. Cross ethnic studies also support the universality of this association. An analysis by Bosch et al. of 3,211 participants from six ethnic groups including Dutch, South Asian Surinamese, African Surinamese, Ghanaian, Turkish, and Moroccan showed that both α- and β-diversity of the GM can predict depressive symptom levels, and that this association does not differ significantly across ethnic groups; microbiome differences accounted for 2.9% to 18% of the inter-ethnic variations in depressive symptoms (Li X. et al., 2022). Importantly, microbiota-depression associations may be influenced by potential confounders such as diet, BMI, medication use, antidepressant exposure, and gastrointestinal or metabolic comorbidities. Although some studies have adjusted for these factors or enrolled antidepressant-naïve participants, residual confounding cannot be fully excluded. Therefore, the reported microbial alterations should be interpreted primarily as depression-associated signatures rather than definitive causal biomarkers.

Beyond changes in microbial diversity, several specific bacterial taxa have been repeatedly associated with depressive disorders. Although the reported microbial signatures vary among cohorts, systematic reviews and large population-based studies have identified several relatively consistent patterns. At the genus level, *Eggerthella*, *Enterococcus*, *Flavonifractor*, *Holdemania*, *Streptococcus*, *Lachnoclostridium*, and *Hungatella* are frequently enriched in individuals with depression or greater depressive symptom severity (Barandouzi et al., 2020; Borkent et al., 2022; McCurry et al., 2024; Nikolova et al., 2021; Radjabzadeh et al., 2022; Zhong et al., 2022), whereas *Faecalibacterium*, *Coprococcus*, *Butyricicoccus*, *Subdoligranulum*, *Fusicatenibacter*, and *Romboutsia* are commonly depleted (Barandouzi et al., 2020; Gao et al., 2023; Guo et al., 2024; Knudsen et al., 2021; Sabarathinam, 2024). Notably, the reduction in several SCFA-producing genera, such as *Faecalibacterium* and *Coprococcus*, may contribute to impaired intestinal barrier function and enhanced inflammatory signaling, whereas enrichment of inflammation-associated genera such as *Eggerthella* and *Enterococcus* may further promote a pro-inflammatory intestinal environment. These findings suggest that depression-associated gut dysbiosis is characterized not merely by altered microbial diversity, but also by reproducible shifts in specific microbial taxa with potential functional consequences for host metabolism and immune regulation. Fecal microbiota transplantation (FMT) experiments have provided further functional evidence supporting the contribution of depression-associated GM to depressive phenotypes. Transplantation of fecal microbiota from patients with major depressive disorder (MDD) into germ-free or microbiota-depleted rodents was sufficient to induce depression- and anxiety-like behaviors, accompanied by disturbances in host amino acid and tryptophan metabolism (Kelly et al., 2016). These effects appear to reflect the collective functional properties of a dysbiotic microbial community rather than the action of a single bacterial species. Conversely, studies using healthy-donor FMT and targeted bacterial supplementation have identified several potentially protective taxa (Zhou et al., 2023). In particular, *Roseburia* was significantly depleted in patients with adolescent depression, whereas healthy-donor FMT restored *Roseburia* abundance and alleviated depressive-like behaviors in chronic restraint stress mice. Subsequent targeted supplementation with *Roseburia* intestinalis further confirmed its functional contribution, increasing brain and colonic 5-HT levels while reducing KYN-pathway metabolites, including quinolinic acid and 3-hydroxykynurenine. These findings suggest that specific depression-associated bacterial taxa may contribute to the regulation of depressive phenotypes through modulation of tryptophan metabolism.

## 4 Regulation of tryptophan metabolism via the gut-brain axis: from core pathways to signaling

### 4.1 The core pathway of tryptophan metabolism: division of labor between host and microbiota

#### 4.1.1 Host-dominant pathways

Within the host organism, Trp is preferentially directed toward 5-HT synthesis and the KYN pathway, which together constitute the core metabolic hub for mood and neuroimmune regulation. Trp is converted to 5-hydroxytryptophan (5-HTP) under the catalysis of TPH, and subsequently decarboxylated to form 5-HT. Central 5-HT is primarily driven by TPH2 and is involved in the fine-tuning of mood, stress responses, and reward pathways; alterations in 5-HT levels are closely associated with mood disorders such as depression and anxiety, as specifically demonstrated in animal models and clinical trials (Deng et al., 2021; El-Haggar et al., 2018). The KYN pathway is the predominant route for Trp degradation *in vivo*, with approximately 95% of free Trp entering this pathway (Correia and Vale, 2022). Trp is first converted to KYN by IDO and tryptophan 2,3-dioxygenase (TDO), and subsequently branches to form 3-hydroxykynurenine (3-HK), QUIN, kynurenic acid (KYNA), nicotinamide adenine dinucleotide (NAD^+^), and various other metabolites (Correia and Vale, 2022). In depression and related inflammatory states, substantial evidence points to a shift of Trp toward the KYN pathway: in patients with major depression, bipolar disorder, and schizophrenia, peripheral Trp and KYN levels are both reduced, whilst the KYN/Trp ratio is generally elevated, suggesting activation of the IDO/TDO pathway and suppression of the 5-HT pathway. Studies have found that inflammatory activation can lead to reduced Trp levels, elevated KYN levels, and an increased KYN/Trp ratio, accompanied by higher depression scores (Marx et al., 2021). KYN itself also exhibits characteristics of an inflammatory mediator: in chronic stress models, KYN levels in the hippocampus are elevated, and it specifically activates the NLRP2 inflammasome in astrocytes, promoting NF-κB-dependent IL-1β production; knockdown of NLRP2 blocks KYN-induced depression-like behavior (Zhang et al., 2020). This suggests that KYN may act as a risk factor driving neuroinflammation and influencing the onset and progression of depression (Figure 1).

**FIGURE 1:**
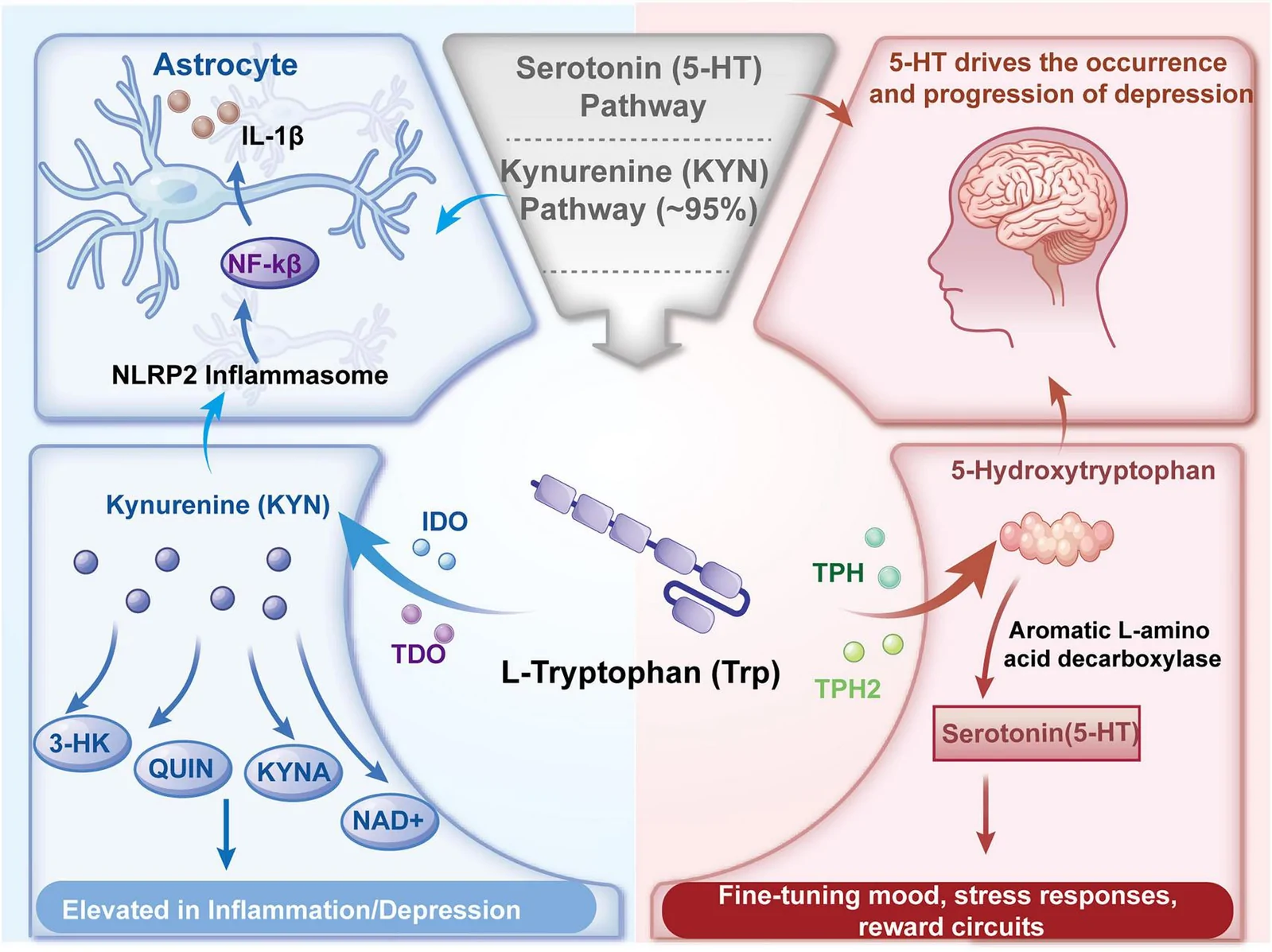
Tryptophan diversion from serotonin to KYN pathway links neuroinflammation to depression via IDO/TDO activation. Trp generates 5-HT via TPH/TPH2, participating in the regulation of mood, stress, and reward; on the right side: approximately 95% of Trp enters the KYN pathway via IDO/TDO, producing KYN and its downstream metabolites (3-HK, QUIN, KYNA, NAD^+^). Under inflammatory/depressive conditions, the KYN pathway is activated (elevated KYN/Trp ratio), and KYN activates the NLRP2 inflammasome in astrocytes, promoting IL-1β production via NF-κB, thereby driving neuroinflammation and depression progression.

#### 4.1.2 Microbiota-specific pathways

Gut microbial pathways also play a crucial role in Trp metabolism. Gut bacteria express tryptophanase (TnaA) to break down Trp into indole, pyruvate, and ammonia; some of the indole produced acts as a signaling molecule within the intestinal lumen, whilst the remainder is absorbed into the bloodstream and sulphated in tissues such as the liver and kidneys to form conjugates such as indoxyl sulphate. In addition to direct indole formation, Trp can also be converted by aromatic amino acid transaminase within the same bacterium or by commensal bacteria into indole-3-pyruvic acid, which is then converted via decarboxylation and dehydrogenation to indole-3-acetaldehyde, subsequently oxidized or reduced to indole-3-acetic acid (IAA) or reduced and elongated to indole-3-propionic acid (IPA) and other derivatives (Konopelski and Mogilnicka, 2022). Importantly, many microbiota-derived indole metabolites function as endogenous ligands of the aryl hydrocarbon receptor (AhR), making AhR a key molecular sensor that translates microbial Trp metabolism into host physiological responses. Representative metabolites, including IAA, ILA, IPA, and IAld, can modulate AhR signaling, although their receptor affinity and biological effects vary according to ligand structure and tissue context. In the intestine, activation of AhR contributes to epithelial barrier integrity, mucosal immune homeostasis, antimicrobial defense, and IL-22-associated epithelial protection, thereby restricting the translocation of microbial products and excessive peripheral inflammatory signaling (Sun et al., 2020). This microbiota-derived indole-AhR signaling axis may also be particularly relevant to the pathogenesis of depression. Gut dysbiosis can alter the abundance of Trp-metabolizing bacteria and consequently change the availability of AhR-active indole metabolites. Impaired or imbalanced AhR signaling may compromise intestinal barrier and immune homeostasis, facilitating systemic inflammation that can subsequently influence neuroimmune responses through the gut-brain axis. Moreover, AhR is expressed in CNS-resident cells, including astrocytes and microglia, where AhR activation by Trp-derived metabolites can modulate inflammatory signaling pathways, including NF-κB and inflammasome-related responses. Emerging experimental evidence further indicates that modulation of the microbiota-indole-AhR axis can influence neuroinflammation, hippocampal neurogenesis, stress susceptibility, and depression-like behaviors. Therefore, microbiota-derived indole metabolites should be regarded not merely as metabolic end products, but as signaling molecules capable of linking gut microbial dysbiosis to depression through AhR-dependent intestinal, immune, and neurobiological mechanisms.

Importantly, the kynurenine, serotonin, and microbial indole pathways are not independent metabolic routes but are dynamically interconnected through their shared dependence on Trp availability. The allocation of Trp among these pathways is jointly determined by substrate availability, host enzyme activity, microbial metabolic capacity, and physiological or pathological signals. In host tissues, TPH directs Trp toward serotonin synthesis, whereas IDO and TDO promote its conversion into KYN. Under inflammatory conditions, cytokine-induced IDO activation can increase Trp flux toward the KYN pathway, while stress-related glucocorticoids may enhance TDO activity, thereby reducing the relative availability of Trp for serotonin synthesis. Meanwhile, gut microorganisms directly consume luminal Trp to generate indole and its derivatives and may also indirectly influence host Trp metabolism by modulating intestinal inflammation and the activity of IDO and TPH. Thus, dietary Trp availability, microbial composition, inflammatory status, and neuroendocrine stress signals collectively shape the dynamic balance among the three pathways. In depression, disruption of this metabolic balance may favor KYN metabolism, reduce serotonergic availability, and alter the production of microbiota-derived indole metabolites.

Overall, depression-associated Trp metabolism is characterized by a disturbed dynamic balance among the kynurenine, serotonin, and microbial indole pathways, rather than by dysregulation of any single metabolic route.: Stress and inflammation, by activating IDO/TDO, drive Trp toward the KYN pathway; this not only impairs 5-HT synthesis but also leads to the accumulation of neurotoxic metabolites, driving glutamate excitotoxicity and neuroinflammation, thereby facilitating the onset and maintenance of depression-like behavior. Simultaneously, gut dysbiosis may reduce or alter the production of microbiota-derived indole metabolites and consequently disrupt AhR-dependent signaling. Such alterations can impair intestinal barrier and immune homeostasis, amplify peripheral inflammation, and subsequently influence neuroimmune responses through the gut-brain axis, thereby contributing to the pathophysiology of depression. Together, these factors constitute the key metabolic basis of depression pathology and lay the foundation for the theoretical framework of “Trp metabolite-mediated gut-brain signaling.” These interconnected metabolic processes are further summarized in Table 1, which provides an overview of the major Trp-derived metabolites, their metabolic origins, biological functions, and potential contributions to the pathogenesis of depression (Table 1).

**TABLE 1:** Key tryptophan-derived metabolites and their functions in depression pathogenesis.

Metabolite	Pathway	Source (host/ microbiota)	Primary function in depression	Key mechanisms	References
Serotonin (5-HT)	Serotonin	Host	Antidepressant	Regulates mood, emotion, and neuroplasticity via 5-HT receptors.	Nikolova et al., 2021; McCurry et al., 2024
Kynurenine (KYN)	Kynurenine	Host	Pro-inflammatory / Bimodal	Activated by IDO/TDO; acts as an inflammatory mediator; can be metabolized to neurotoxic or neuroprotective metabolites.	Zhong et al., 2022; Radjabzadeh et al., 2022
Quinolinic Acid (QUIN)	Kynurenine	Host (Microglia)	Neurotoxic	NMDA receptor agonist; induces excitotoxicity, oxidative stress, and astrocyte apoptosis.	Zhong et al., 2022
Kynurenic Acid (KYNA)	Kynurenine	Host (Astrocytes)	Neuroprotective	Glutamate receptor antagonist; blocks NMDA receptors; provides synaptic modulation and neuroprotection.	Radjabzadeh et al., 2022
Indole	Indole	Microbiota	Signaling Molecule	Regulates intestinal homeostasis; acts as a signaling molecule in the gut lumen.	Barandouzi et al., 2020
Indole-3-propionic acid (IPA)	Indole	Microbiota	Antidepressant / Neuroprotective	Potent free radical scavenger; activates AhR and GPR35; reduces inflammation and oxidative stress; crosses BBB.	Barandouzi et al., 2020; Zhang M. et al., 2021; Hu et al., 2022
Indole-3-acetic acid (IAA)	Indole	Microbiota	Antidepressant	Activates AhR; modulates central 5-HT system; reduces neuroinflammation.	Barandouzi et al., 2020; Salminen, 2023
Indole-3-carboxaldehyde (I3C)	Indole	Microbiota	Antidepressant	Activates AhR-SOCS2-NF-κB/NLRP3 pathway; suppresses neuroinflammation and promotes hippocampal neurogenesis.	Haroon et al., 2020

### 4.2 Tryptophan metabolite-mediated gut-brain signaling and neuroregulation

Emerging evidence suggests that the involvement of Trp metabolism in depression may extend beyond a direct microbiota-gut-brain pathway and instead operate within a broader inter-organ metabolic network. Trp metabolites are generated, transformed, and cleared across multiple tissues, such that their circulating availability reflects coordinated interactions among the GM, liver, immune system, peripheral tissues, and brain. In particular, the liver represents a major metabolic hub because TDO-mediated Trp catabolism substantially contributes to systemic KYN production, whereas peripheral inflammation can further enhance IDO-dependent Trp degradation. These processes may alter the circulating KYN pool and consequently influence the amount of KYN available for transport into the brain and subsequent conversion into neuroactive metabolites (Tang X. et al., 2025). From this perspective, the GM may act as an upstream regulator of Trp availability and microbial indole production, while peripheral organs further modify the composition and bioavailability of circulating Trp metabolites before they influence the central nervous system. Other tissues, including skeletal muscle and the kidney, may additionally regulate peripheral KYN conversion or metabolite clearance. Thus, depression-associated abnormalities in Trp metabolism may reflect dysfunction of a distributed inter-organ network rather than an isolated gut-to-brain pathway. This emerging framework may help explain inter-individual differences in Trp metabolic profiles and provide additional opportunities for mechanism-based and personalized interventions.

#### 4.2.1 Regulation of the blood-brain barrier and central inflammation by gut microbiota metabolites

The GM metabolizes Trp into various indole compounds; after these metabolites enter the systemic circulation via the portal vein, they are able to cross the blood-brain barrier (BBB) and activate AhR on cerebral microvascular endothelial cells. AhR activation induces the expression of downstream CYP1 family enzymes and immunomodulatory factors, thereby participating in the regulation of cerebral vascular permeability and central inflammatory responses, and influencing BBB integrity and microglial activation in states of neuritis (Salminen, 2023). In brain parenchyma, microbiota-dependent Trp metabolism alters the expression patterns of the key rate-limiting enzymes TPH2 and IDO1 by regulating the competitive balance between the 5-HT synthesis and KYN pathways (Zhou et al., 2023). Studies have shown that Trp and 5-HT levels in the hippocampus and prefrontal cortex of germ-free mice are significantly elevated, accompanied by upregulation of TPH2 expression; this suggests that in the absence of GM, brain Trp metabolism is more directed toward the 5-HT synthesis pathway (Lukić et al., 2019). Conversely, under conditions of stress and dysbiosis, hippocampal Trp metabolism shifts toward the KYN pathway, manifested by a significant increase in IDO1 expression, a decrease in TPH2 expression, and concurrent increases in KYN levels and the KYN/Trp ratio, whilst 5-HT and its metabolite levels decrease (Deng et al., 2021). Interventions targeting the GM, such as rifaximin administration, can reverse the Trp-KYN metabolic imbalance in the hippocampus, reducing IDO1 expression, increasing TPH2 levels, and restoring 5-HT levels, thereby improving depression-like behavior (Cheng et al., 2023). This suggests that Trp metabolites derived from the GM are key factors in regulating the 5-HT/KYN balance in the brain (Figure 2).

**FIGURE 2:**
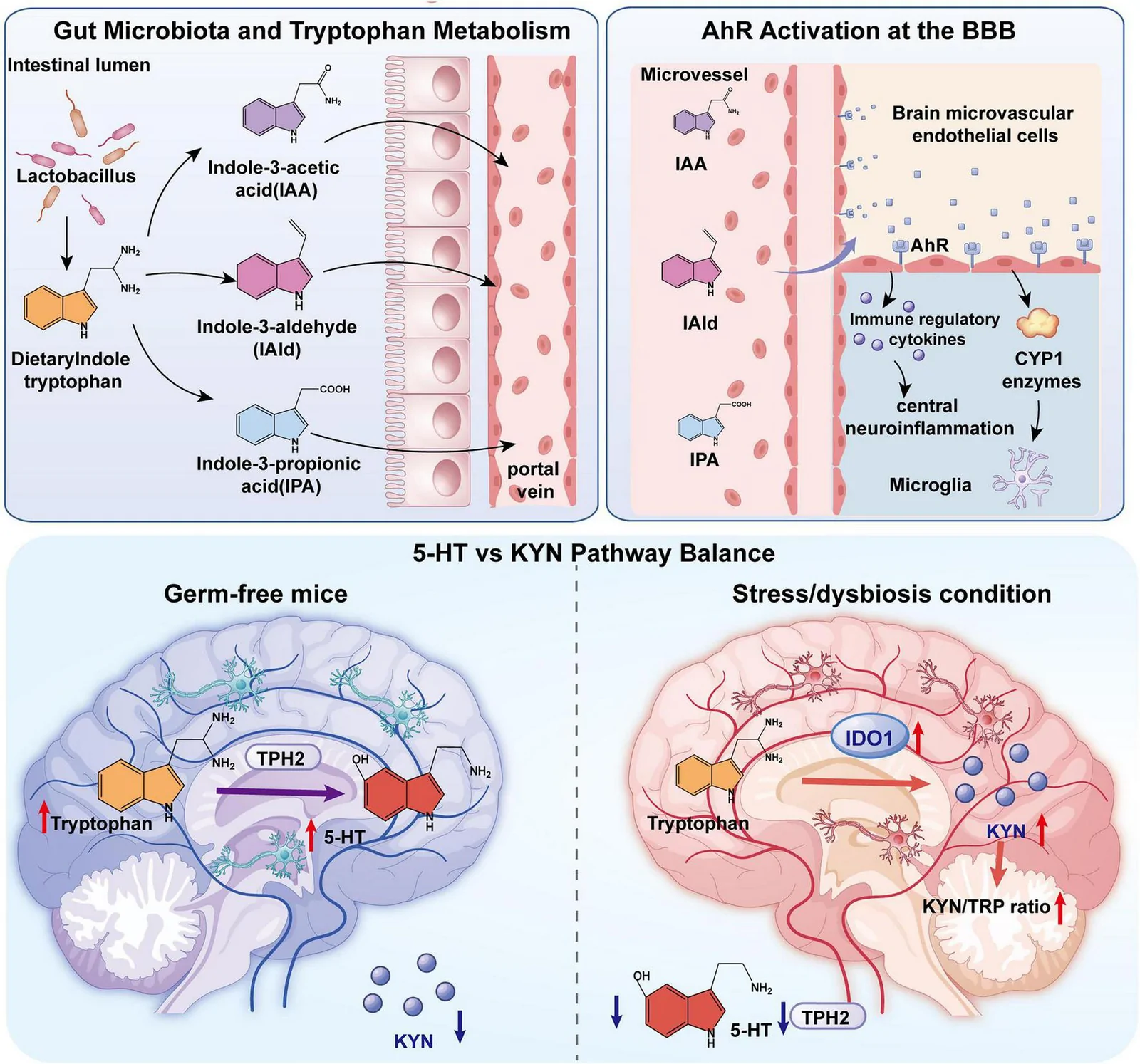
Gut microbiota-derived tryptophan metabolites regulate BBB function and central inflammation via AhR and 5-HT/KYN pathways. Gut microbiota metabolize Trp into indole compounds (IAA, IAld, IPA), which enter the portal circulation and cross the BBB, where they activate the aryl hydrocarbon receptor (AhR) on cerebral microvascular endothelial cells, inducing the expression of CYP1 enzymes and immunomodulatory factors, thereby participating in the regulation of BBB permeability and neuroinflammation. Meanwhile, microbiota-dependent Trp metabolism influences the balance of rate-limiting enzymes in the brain: in germ-free mice, TPH2 is upregulated and 5-HT is elevated, favoring the 5-HT pathway; under stress or dysbiosis, IDO1 is upregulated and TPH2 is downregulated, shifting metabolic flux toward the KYN pathway (KYN/Trp ratio increases, 5-HT decreases). This imbalance can be reversed by microbiota-targeted interventions, improving depression-like behaviors.

#### 4.2.2 Regulation of the 5-HT/KYN balance in the brain by tryptophan metabolism

Changes in Trp and its metabolites can directly regulate neurotransmitter signaling. Some Trp is converted into 5-HTP under TPH catalysis, which is subsequently converted into 5-HT. As a key neurotransmitter, 5-HT is involved in the regulation of adaptive responses in the CNS. 5-HT is transported via the vesicular monoamine transporter 2 (VMAT2) and stored in synaptic vesicles; upon arrival of a nerve impulse, it is released into the synaptic cleft via exocytosis. Although central 5-HT levels are relatively low, owing to its extensive neural projection network, 5-HT plays a central regulatory role in higher-order neural activities such as emotional regulation, cognitive function, anxiety responses, learning and memory, reward mechanisms, and the sleep-wake cycle. The termination of 5-HT signaling relies primarily on the reuptake process mediated by the 5-HT transporter (SERT). SERT utilizes the electrochemical gradient of sodium and chloride ions to transport 5-HT from the synaptic cleft back into the nerve terminal or for uptake by neighboring glial cells. Once inside the cytoplasm, 5-HT can be metabolized by monoamine oxidase (MAO) into 5-hydroxyindoleacetic acid (5-HIAA), which is ultimately excreted via the kidneys. This delicate balance between synthesis, transport, and metabolism forms the basis for maintaining normal neural circuit function; disruption at any stage may trigger depression (Miler et al., 2019).

#### 4.2.3 The vicious cycle of neurotransmitter signaling, the HPA axis, and tryptophan metabolism

At the genetic level, alterations in the HPA axis, such as impaired glucocorticoid receptor expression, can affect 5-HT neurotransmission. Conversely, knockout of SERT (5-HTT) alters the HPA axis’s dependence on stress responses. Trp is degraded via the KYN pathway and regulates neuroinflammation across the BBB. Following activation by pro-inflammatory cytokines or glucocorticoids, Trp is degraded to KYN via the KYN pathway, which is subsequently metabolized to QUIN in microglia and macrophages. QUIN is gliotoxic; it induces astrocyte apoptosis and modulates glutamatergic neurotransmission by activating N-methyl-D-aspartate (NMDA) receptors, thereby exerting neurotoxic and oxidative stress effects (Marx et al., 2021). In astrocytes, KYN is converted into KYNA, which acts as a glutamate receptor antagonist by binding to the glycine binding site, thereby exerting synaptic modulatory effects and providing neuroprotective functions (Zhang et al., 2020). Studies have shown that, compared with control groups and depressed patients who died from other causes, depressed suicide victims exhibited reduced Trp levels alongside elevated KYN and QUIN levels (Messaoud et al., 2019). Under chronic stress, the KYN pathway forms a vicious cycle with the HPA axis: stress drives a shift in KYN metabolism toward KYNA, disrupting the negative feedback regulation of the HPA axis; simultaneously, excessive HPA axis activation induces IDO via glucocorticoids, causing Trp metabolism to favor the production of neurotoxic metabolites. This suggests that the interaction between Trp metabolism and the HPA axis is particularly relevant to depression. Trp supplementation restores serum Trp and 5-HTP levels in mice to normal, significantly restores the number of newborn neurons in the hippocampus, and improves BBB integrity, indicating that Trp is sufficient to block the spread of peripheral inflammation to the CNS, re-establish excitatory synaptic transmission, and alleviate depressive behavior (Zhang et al., 2025; Figure 3).

**FIGURE 3:**
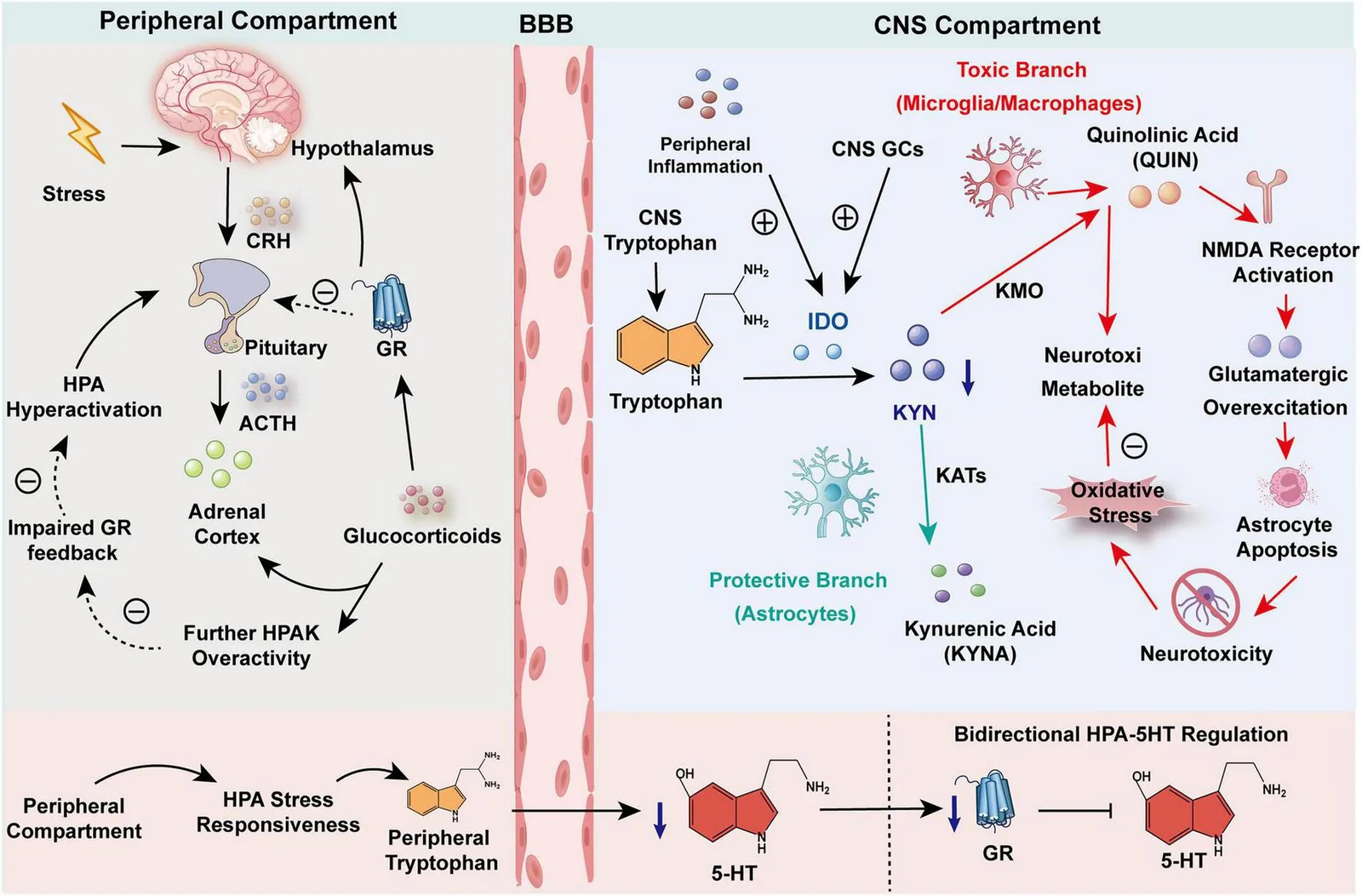
HPA axis dysfunction and tryptophan-kynurenine metabolism interact to drive neuroinflammation and depression via QUIN neurotoxicity. The HPA axis/pro-inflammatory cytokines activate IDO, driving Trp degradation via the KYN pathway: QUIN is generated in microglia/macrophages, activating NMDA receptors, inducing astrocyte apoptosis and oxidative stress, resulting in neurotoxicity; KYNA is generated in astrocytes, antagonizing glutamate receptors, exerting neuroprotection. Under chronic stress, the KYN pathway and the HPA axis form a vicious cycle (stress shifts KYNA, while HPA overactivation further promotes IDO), exacerbating metabolic imbalance. In the brains of depressed suicide victims, Trp is decreased and KYN/QUIN are increased. Trp supplementation can restore 5-HTP, hippocampal neurogenesis, and BBB integrity, alleviating depression.

#### 4.2.4 Sex differences in tryptophan metabolism and depression-related regulation

These differences may be influenced by sex hormones through effects on gut microbial composition, serotonergic signaling, and Trp-metabolizing enzymes.

Available evidence suggests that sex may influence Trp metabolism and its relationship with mood regulation, although the underlying mechanisms remain incompletely defined and appear to vary across experimental models and physiological contexts. Preclinical studies indicate that females may exhibit a greater tendency toward KYN-pathway activation under certain inflammatory or stress conditions, whereas males may show relatively greater engagement of microbiota-dependent indole metabolism (Bardhan et al., 2024). Sex-dependent differences have also been reported in serotonergic stress adaptation: males may rely more strongly on presynaptic 5-HT1A receptor regulation in the dorsal raphe nucleus, whereas females may exhibit greater postsynaptic 5-HT1A responsiveness in the hippocampus (Philippe et al., 2022; Xu et al., 2025). However, the available evidence is derived largely from animal or mechanistic studies, and findings are not always consistent across species, brain regions, or experimental conditions. Human studies directly linking sex-specific Trp metabolic profiles to depressive phenotypes remain relatively limited. Therefore, sex should currently be regarded as an important biological modifier of the microbiota-Trp-depression axis rather than as a determinant of a fixed sex-specific metabolic pattern.

#### 4.2.5 Critical perspective and research implications of the mechanistic model

The integrated model of 5-HT/KYN/Indole pathways provides a robust molecular framework for understanding depression. However, it is crucial to remember that these mechanisms are often studied in isolation or in highly controlled animal models. In the complex pathophysiology of human depression, these pathways likely interact with other major systems, including the hypothalamic-pituitary-adrenal (HPA) axis and the immune system. Moreover, current microbiome evidence does not support a single disease-specific causal microbial signature; instead, findings are largely correlational and require better-controlled longitudinal studies. A key limitation is that the relative contributions of the serotonin, kynurenine, and microbial indole pathways in individual patients remain uncertain, and longitudinal metabolic-flux studies are needed to clarify their dynamic interplay. Future research must move beyond correlating metabolites with symptom severity and instead employ causal inference methodologies, such as Mendelian randomization in large human cohorts or targeted pathway-specific interventions in well-designed clinical trials. For therapeutic interventions, it is essential to distinguish promising mechanistic and preclinical findings from clinically validated approaches; randomized clinical trials, standardized biomarkers, safety evaluation, and biomarker-guided patient stratification are required before translation. Furthermore, identifying reliable biomarkers of Trp metabolism that can be measured repeatedly and non-invasively (e.g., from a simple blood draw) is the next critical step to translate this mechanistic knowledge into actionable clinical tools for diagnosis, prognosis, and treatment monitoring.

## 5 Intervention strategies based on the tryptophan-microbiota axis

Intervention strategies for regulating depression via the Trp-microbiota axis can be broadly categorized into three types: dietary interventions, microbiota-targeted therapies (probiotics, antibiotics, traditional Chinese medicine), and direct supplementation with Trp metabolites (Figure 4).

**FIGURE 4:**
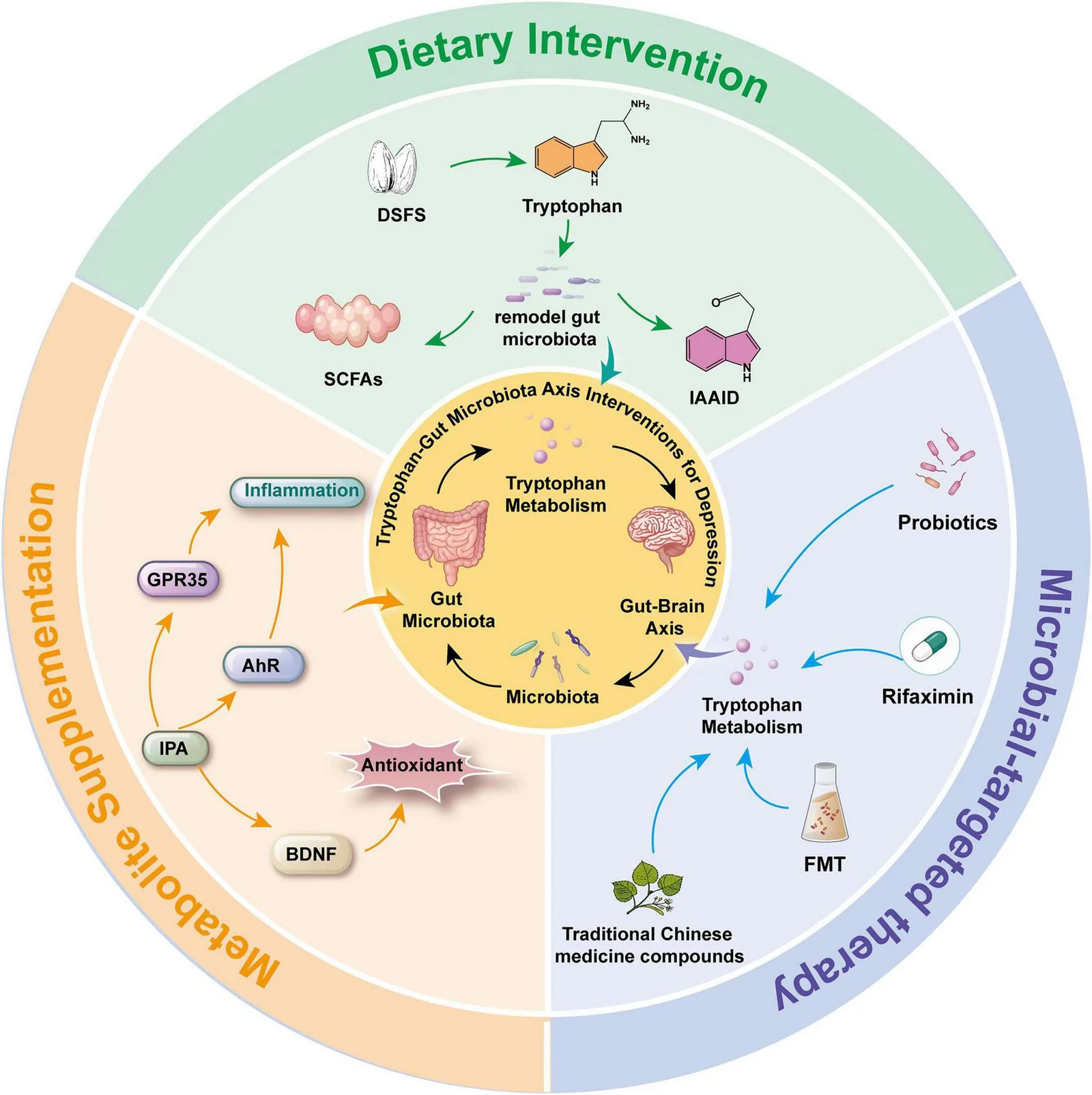
Tryptophan-gut microbiota axis interventions for depression. Intervention strategies for regulating depression via the Trp-microbiota axis can be broadly categorized into three types: dietary interventions, microbiota-targeted therapies (probiotics, antibiotics, traditional Chinese medicine), and direct supplementation with Trp metabolites.

### 5.1 Dietary interventions

Tryptophan-rich, defatted and chlorogenic acid-removed sunflower seeds (DSFS) alleviate depression by reshaping the GM, increasing levels of beneficial metabolites such as indole-3-acetaldehyde, and activating the gut-brain axis. This mechanism of action stems primarily from the high Trp content of sunflower seed protein. DSFS not only provides abundant Trp as a precursor for 5-HT synthesis, but also improves the intestinal microenvironment by removing antinutritional factors (such as chlorogenic acid) through a specialized processing technique, thereby enhancing the bioavailability of protein and Trp (Lu et al., 2022). Studies have shown that DSFS can significantly alter GM composition, promoting the proliferation of specific Trp-metabolizing bacterial communities. These microorganisms convert Trp into indole-3-acetaldehyde (IAAld) (Lu et al., 2022). IAAld and its downstream metabolites (such as IAA) are able to cross the BBB, modulate the central 5-HT system, alleviate inflammatory responses, and thereby improve depression-like behavior (Lu et al., 2021).

In addition to direct Trp supplementation, certain dietary components can improve Trp metabolism by modulating the GM, thereby alleviating depressive symptoms. For example, dietary fiber and polysaccharides, as important substrates for the GM, are degraded to produce SCFAs, including butyrate and propionate (Li Y. et al., 2023). These SCFAs have multiple effects: on the one hand, they enhance gut barrier function, preventing inflammatory factors from entering the bloodstream and triggering neuroinflammation; on the other hand, they can directly cross the BBB to participate in regulating neurotransmitter synthesis and release, improving neuroplasticity and exerting a positive influence on depressive symptoms (Cheng et al., 2024). Furthermore, dietary fiber promotes the shift of Trp metabolism from the KYN pathway to the indole pathway, exerting anti-inflammatory, antioxidant, and mood-regulating effects, thereby alleviating depressive symptoms (Liu et al., 2022).

### 5.2 Microbial-targeted therapies

#### 5.2.1 Probiotics

Modulating the human microbiome to influence Trp metabolism is another intervention strategy for depression. Supplementation with probiotics such as Lactobacillus can help restore intestinal Trp metabolic homeostasis, enhance intestinal barrier function, and alleviate intestinal inflammation and depression. Furthermore, probiotics can regulate GM balance, inhibit pathogenic bacterial overgrowth, and restore the abundance of other beneficial bacteria, thereby promoting the conversion of Trp into beneficial metabolites (Tian et al., 2022). Studies have shown that probiotic-mediated Trp metabolism can inhibit inflammasome activation, reduce the release of pro-inflammatory factors, and alleviate neuroinflammation, thereby maintaining intestinal epithelial cell homeostasis at the molecular level (Wang J. X. et al., 2025).

#### 5.2.2 Antibiotics

Rifaximin is an oral, non-absorbable antibiotic with extremely low bioavailability that primarily exerts local antibacterial effects within the gut; it is already used clinically to treat hepatic encephalopathy and irritable bowel syndrome. Recent preclinical studies have shown that rifaximin exerts a significant protective effect against depression-like behavior. Research has found that chronic unpredictable mild stress (CUMS) can induce gut dysbiosis and, via the “microbiota-gut-brain axis,” lead to disturbances in hippocampal Trp metabolism (Deng et al., 2021). Rifaximin significantly improves behavioral outcomes in CUMS-exposed rats, including sucrose preference, open-field activity, and spatial memory in the water maze, whilst simultaneously correcting GM dysbiosis and restoring the expression balance of IDO1 and TPH2 in the hippocampus (Cheng et al., 2023). Furthermore, rifaximin increases the relative abundance of SCFA-producing bacteria, namely Ruminococcaceae and Lachnospiraceae, whose abundance is positively correlated with brain butyrate levels. This mechanism promotes the transition of microglia from a pro-inflammatory to an anti-inflammatory phenotype, thereby protecting hippocampal neurogenesis. Clinical studies have also confirmed that rifaximin, when used to treat patients with small intestinal bacterial overgrowth, can simultaneously improve anxiety and depressive symptoms as well as abnormalities in Trp metabolism (Chojnacki et al., 2022). Other antibiotics, such as minocycline, have similarly demonstrated antidepressant effects in the CUMS mouse model; they suppress neuroinflammation, modulate the microbiota, and increase the abundance of Lachnospiraceae and plasma butyrate levels in highly anxious rats (Schmidtner et al., 2019). Although these findings support the potential of rifaximin to modulate the microbiota-Trp axis, the mechanistic evidence linking rifaximin to restoration of the central 5-HT/KYN balance is derived predominantly from rodent models. Current human evidence is indirect and largely comes from patients with gastrointestinal disorders rather than depression-specific randomized controlled trials. Therefore, further clinical studies incorporating psychiatric outcomes together with microbiome and Trp-metabolomic profiling are required. Antibiotic-based microbiota modulation also raises concerns regarding antimicrobial resistance and disruption of commensal microbial communities, particularly with prolonged or repeated use. These potential risks should be carefully balanced against therapeutic benefits in future clinical studies.

#### 5.2.3 Traditional Chinese medicine formulations

Some traditional Chinese medicine formulations also exert antidepressant effects by improving microbial composition and Trp metabolism. For example, the Shu Gan He Wei Formula can improve GM structure, increase the abundance of beneficial bacteria, and repair the intestinal barrier, thereby promoting microbiota-derived Trp metabolism; its metabolites effectively regulate hippocampal neuronal autophagy homeostasis by activating AMPK and inhibiting the mTOR signaling pathway, thus reversing chronic stress-induced depression-like behavior (Yue et al., 2024). Concurrently, Si Ni San has been shown to correct Trp metabolic dysregulation in fecal metabolomics under chronic stress conditions and, via the PI3K/Akt/mTOR pathway, prevent corticosterone-induced excessive neuronal autophagy whilst enhancing the expression of brain-derived neurotrophic factor (BDNF), thereby exerting synergistic neuroprotective and anti-inflammatory effects (Zhang M. et al., 2021).

#### 5.2.4 Fecal microbiota transplantation

FMT has emerged as an experimental strategy for broad restoration of gut microbial communities. Although compelling preclinical studies support its ability to modify depression-related behaviors and Trp metabolism, its therapeutic application in patients with depression remains at an early clinical stage. In animal studies, whether healthy donor microbiota is transplanted into depression-prone or chronic stress model animals, or microbiota from depressed patients or adolescents with depression is transplanted into germ-free or antibiotic-treated animals, synchronous changes in behavioral phenotypes and Trp metabolism can be observed: FMT from healthy donors to depression models significantly alleviates depression-like behavior, increases hippocampal or brain 5-HT levels, reduces inflammatory cytokines, and is accompanied by a rebalancing of enzyme expression and metabolite profiles associated with the Trp-KYN pathway, suggesting that the “microbiota-gut-brain axis” corrects the distribution of Trp toward the dual 5-HT/KYN pathways (Cai et al., 2022; Hu et al., 2022). Conversely, transplanting the GM of depressed patients into germ-free or microbiota-depleted rats can induce anhedonia and anxiety-like behavior in the recipients, accompanied by peripheral Trp-KYN metabolic abnormalities, suggesting that the depression-associated microbiota itself possesses a “transferable” Trp metabolic imbalance and depressive phenotype (Kelly et al., 2016; Ruszkowski et al., 2025). In other disease models, such as autism spectrum disorder, GM from affected children can similarly reshape the expression of Trp and 5-HT metabolic enzymes in germ-free mice and induce behavioral abnormalities, further corroborating the causal relationship between GM, Trp metabolism, and brain function (Xiao et al., 2021).

However, evidence remains limited regarding the translation from animal models to clinical practice: current FMT studies in individuals with depression or comorbid depression and anxiety are predominantly small-sample case reports or cohorts with co-morbid somatic conditions such as functional constipation or irritable bowel syndrome. Although improvements in depressive symptoms and quality of life, changes in neurotransmitter levels such as 5-HT, and GM composition have been observed, the study populations are often heterogeneous and follow-up periods relatively short; there is still a lack of large-sample, long-term randomized controlled trials specifically targeting depressive disorders to systematically evaluate efficacy and relapse rates (Chinna Meyyappan et al., 2020; Doll et al., 2022; Yang et al., 2023). At the same time, existing systematic and narrative reviews generally indicate that FMT remains in early exploratory stages in the field of mental disorders; most evidence stems from preclinical studies, and there are as yet no unified standards regarding optimal donor characteristics, routes of administration, treatment duration, or safety. Furthermore, the specific regulatory mechanisms of Trp metabolic pathways in clinical populations require further elucidation through multi-omics approaches and precision stratification designs (Chang M. et al., 2024; Meng et al., 2022; Yadegar et al., 2024). Two ongoing FMT trials (NCT04805879, NCT06692361) are assessing whether microbiota restoration improves depressive symptoms, alongside changes in microbial and metabolic profiles. Therefore, based on animal studies and preliminary clinical observations, there is strong theoretical and experimental support for the notion that FMT improves depression-related behaviors by reshaping the GM-Trp metabolic network.

Safety also remains a major concern for the clinical application of FMT. Because FMT involves the transfer of a complex and incompletely defined microbial community, there is a potential risk of transmitting pathogenic or antimicrobial-resistant microorganisms, highlighting the importance of rigorous donor screening, pathogen testing, and long-term safety surveillance. Therefore, although current preclinical and preliminary clinical evidence is encouraging, high-quality clinical trials and mechanistic studies are still required before FMT can be developed into a standardized treatment strategy for depression.

### 5.3 Metabolite supplementation

Trp and its specific indole derivatives have demonstrated significant potential in interventions for depression, primarily due to their unique mechanisms of regulating inflammation and oxidative stress via the gut-brain axis.

In recent years, 5-HTP, as the direct precursor of 5-HT, has garnered substantial interest in the treatment of depression (Maffei, 2020). Although 5-HTP theoretically possesses the potential to ameliorate depressive symptoms by elevating central 5-HT levels, its clinical utility has been constrained by pharmacokinetic limitations, including a short plasma half-life after oral administration and extensive peripheral metabolism, which impede its efficient penetration across the blood-brain barrier (BBB) and accumulation at central targets. To overcome this challenge, multiple studies have attempted to improve its delivery efficiency through sustained-release formulations (Jacobsen et al., 2016) and nanocarriers, such as near-infrared light-responsive artificial synaptic vesicles (Chang P. et al., 2024). In response to this issue, an ongoing Phase II trial (NCT05895747) is evaluating the efficacy of 5-HTP combined with creatine in patients with treatment-resistant MDD who are non-responders to SSRIs/SNRIs, and has innovatively incorporated plasma serotonin and neuroimaging biomarkers as measures of target engagement, with the aim of exploring strategies to enhance the central effective concentration of 5-HTP while avoiding peripheral adverse effects and achieving targeted regulation.

In terms of inflammation regulation, IPA significantly influences signaling between the GM and the brain by acting on key receptors such as G protein-coupled receptor 35 (GPR35) and AhR. Specifically, it suppresses the production of pro-inflammatory cytokines (such as IL-1β) and improves intestinal barrier function, thereby blocking the transmission of peripheral inflammatory signals to the CNS. This mechanism is of great significance for alleviating the chronic low-grade inflammatory state commonly observed in depressed patients (Chen et al., 2022).

With regard to oxidative stress, IPA, as a potent free radical scavenger, is able to cross the BBB and exert antioxidant effects directly within the CNS. It reduces oxidative stress levels in brain tissue, protects neurons from damage, and simultaneously upregulates BDNF expression. These effects collectively promote improvements in neural plasticity and mood in depressed patients (Owe-Larsson et al., 2025). Given its favorable safety profile and biological activity, IPA has now entered the clinical trial phase. Preliminary research findings indicate that the substance is well tolerated and exhibits biological activity in healthy adults. Furthermore, oral Trp supplements, as precursors to IPA, have also been shown to exert indirect antidepressant effects by increasing IPA levels *in vivo*.

Additionally, Directly modulating the host’s Trp metabolic enzymes represents a promising avenue. Pharmacological inhibition of indoleamine 2,3-dioxygenase (IDO), the rate-limiting enzyme of the KYN pathway, has shown antidepressant potential in preclinical models by restoring the Trp/5-HT balance and reducing neurotoxic KYN metabolites (da Silva Dias et al., 2016). While clinical trials of specific IDO inhibitors are still in early stages for depression, their application in oncology (e.g., epacadostat) provides a safety and pharmacokinetic framework that could be repurposed (Fujiwara et al., 2022). Future research should prioritize the development of brain-penetrant IDO/TDO inhibitors and evaluate their efficacy in patient subgroups with high inflammatory biotypes (e.g., high CRP).

The heterogeneity of Trp metabolism across individuals underpins the need for a precision medicine approach. Stratifying patients by their baseline metabolic profile (e.g., high KYN/Trp ratio vs. low 5-HT levels), inflammatory status (e.g., CRP levels), and gut microbiome composition (e.g., enterotype) could guide therapy selection. For instance, patients with a hyperactive KYN pathway might benefit most from IDO inhibitors or AhR agonists, while those with a deficit in 5-HT synthesis might respond better to Trp or IPA supplementation. This ‘stratified psychiatry’ model represents the future of Trp-based interventions (Table 2).

**TABLE 2:** Evidence level for tryptophan-microbiota axis interventions in depression.

Intervention	Primary evidence base	Current clinical status	Key translational gaps/limitations	References
Dietary interventions	Preclinical (rodent)	Early-stage translational	Need large-scale, dietary-controlled human trials; variable bioavailability.	Lu et al., 2022; Lu et al., 2021
Probiotics	Preclinical and clinical	Some RCTs show moderate benefit	Strain-specific effects; lack of robust, replicable outcomes across trials; regulatory hurdles.	Tian et al., 2022; Wang J. X. et al., 2025
Antibiotics	Preclinical (mainly)	Approved for other indications (e.g., IBS), repurposing in early trials	Risk of disrupting beneficial microbes; may exacerbate dysbiosis with long-term use; not a sustainable depression therapy.	Schmidtner et al., 2019; Chojnacki et al., 2022; Deng et al., 2021
Traditional Chinese medicine	Preclinical	Limited clinical validation	Mechanistic complexity; active ingredients unknown; need for rigorous clinical trials.	Yue et al., 2024; Zhang M. et al., 2021
Fecal microbiota transplantation (FMT)	Preclinical (strong), clinical (weak)	Small case-series/cohorts; no RCTs for depression alone	Risk of infections, ethical concerns, donor variability, durability, standardization.	Hu et al., 2022; Ruszkowski et al., 2025; Yang et al., 2023
Engineered bacteria	Preclinical (proof-of-concept)	Not yet in clinical trials	Biosafety, environmental containment, potential for horizontal gene transfer, high regulatory bar.	Fatimi et al., 2025; Gao et al., 2025
AhR ligand/supplementation	Preclinical (strong), clinical (early)	Clinical trial for IPA in healthy adults	Ligand specificity, dose-response, target tissue selectivity, off-target toxicity.	Chen et al., 2022; Owe-Larsson et al., 2025

As summarized in Table 2, the evidence base for these interventions is heavily weighted toward preclinical animal models. While findings from these studies are promising, they must be interpreted with caution due to substantial differences in gut physiology, metabolic rates, and microbiota composition between rodents and humans. Several interventions, such as FMT and engineered bacteria, face significant practical challenges related to safety, standardization, and long-term efficacy before they can be considered for clinical application. The striking disconnect between the robust effects seen in mice and the often modest effects seen in human trials underscores the urgent need for methodologically rigorous, large-scale randomized controlled trials in well-characterized patient populations (Fatimi et al., 2025).

## 6 Challenges and future directions

A major challenge in this field is the substantial gap between mechanistic evidence from animal models and demonstrated therapeutic efficacy in humans. Many microbiota-targeted interventions discussed above, including FMT, rifaximin-mediated microbiota modulation, specific bacterial supplementation, and engineered probiotics, have shown promising effects on depressive-like behaviors and Trp metabolism primarily in rodents. In contrast, human studies remain relatively limited and are often characterized by small sample sizes, heterogeneous populations, variable intervention protocols, and inconsistent microbiome or metabolomic endpoints. Accordingly, preclinical efficacy should not be interpreted as evidence of established clinical benefit, and well-designed human trials are required to determine whether modulation of the microbiota-Trp axis translates into clinically meaningful antidepressant effects. Current evidence on the microbiota-Trp-depression axis remains heterogeneous across populations, sexes, microbial strains, experimental models, and analytical approaches. Among these sources of heterogeneity, population differences, strain specificity, and insufficient dynamic metabolite monitoring represent particularly important bottlenecks for clinical translation.

### 6.1 Bottlenecks in clinical translation

#### 6.1.1 Population heterogeneity

Existing clinical studies are largely confined to specific age groups or regions; for example, the sample size for adolescent depression cohorts is relatively small, limiting the generalisability of findings (Zhou et al., 2023). Although large-scale metabolomics studies have identified associations between alterations in energy and lipid metabolism in depressed patients and the GM (e.g., *Clostridiales*), coverage of the Trp pathway remains insufficient, making it difficult to disentangle the contributions of factors such as age, sex, and diet (Lin et al., 2023). Stress, inflammation, and sex hormones can reshape Trp flux, with gender differences observed; however, systematic data on transgender individuals, endocrine status, and comorbidities are lacking (Amin et al., 2023; Lai et al., 2021). Longitudinal integrated studies examining the relationship between diet, the microbiota, Trp metabolism, and depressive outcomes are still scarce (Clerici et al., 2025; Correia and Vale, 2022).

#### 6.1.2 Strain specificity

In animal studies, FMT from healthy adolescents and colonization by *Roseburia* can improve depressive behavior by upregulating TPH1/TPH2 and inhibiting IDO1 and 3-HAO (Lukić et al., 2019), and *Parabacteroides* may exert a “probiotic-like” effect by regulating the urea cycle in dogs (Kelly et al., 2016). In humans, however, even genera proposed as probiotics in animal models can be increased in depressed patients (Lai et al., 2021; Park et al., 2023), a pattern accompanied by downregulation of tryptophan synthase activity. These findings suggest that the effects of individual strains are highly dependent on the host environment and microbiota background. Moreover, the effect of a single strain within the complex gut ecosystem is easily modified by microbial interactions, leading to significant translational uncertainty (Tian et al., 2020; Tian et al., 2021; Tian et al., 2022).

A major translational bottleneck is the strain- and context-specificity of microbial effects, which can lead to paradoxical findings. For example, although some Parabacteroides species have been proposed as potential probiotics in animal models (Kelly et al., 2016), other studies have reported an increased abundance of Parabacteroides in depressed patients (Lai et al., 2021). This contradiction likely arises from species- or even strain-level functional differences, as well as from host genetic and dietary backgrounds. Similarly, although Bifidobacterium is often considered a “beneficial” genus, elevated levels of certain Bifidobacterium strains have been reported in some MDD cohorts (Park et al., 2023), suggesting that not all strains are protective or that the host environment can redirect their metabolic output. These discrepancies highlight that a single bacterial genus cannot be universally classified as “good” or “bad” without precise functional characterization and consideration of the broader microbial ecosystem.

#### 6.1.3 Insufficient dynamic detection techniques

Animal experiments have enabled simultaneous detection of multiple Trp metabolites in the gut, blood, and brain, revealing that chronic stress activates the toxic KYN pathway in the colon, a process that can be partially reversed by IDO inhibitors (Deng et al., 2021; Zhao R. et al., 2022). However, most clinical studies rely solely on a single measurement of the Kyn/Trp ratio in peripheral blood and a limited number of fecal metabolites to infer central status (Haroon et al., 2020; Messaoud et al., 2019; Reininghaus et al., 2024). The wide variety and low concentrations of microbial metabolites pose challenges for detection sensitivity and standardization, making it difficult to integrate data across centers (Liao et al., 2021; Shaffer et al., 2022); furthermore, the lack of real-time or long-term dynamic monitoring technologies makes it difficult to address clinical questions such as the timing of drug administration. Although the KYN/Trp ratio and related Trp metabolites show potential as mechanistic or stratification biomarkers, their diagnostic and prognostic value in depression has not yet been sufficiently validated for routine clinical use. Further prospective studies with standardized sampling and analytical methods are required before these biomarkers can be incorporated into psychiatric practice.

### 6.2 The need for multi-omics integration

Multi-omics integration and population stratification studies represent key areas of development in the field of Trp metabolism. Through the combined application of metagenomics, metabolomics, and neuroimaging, it is possible to gain a deeper understanding of the individualized GM-Trp-depression mechanism. Multi-omics studies combine gut metagenomic data with serum or fecal metabolomic data, and correlate these with brain structural and functional imaging, thereby revealing the close relationship between microbe-derived neuroactive metabolites (including KYN and other amino acids, B vitamins, bile acids, etc.) and specific brain network functional connectivity, grey matter volume, and white matter integrity, ultimately constructing the complete “microbiota-metabolism-brain connectivity-symptoms” framework (Liu et al., 2025; Zhao H. et al., 2022). Future research could focus on the Trp-KYN metabolic pathway in cohorts of depressed patients, utilizing fecal metagenomic techniques to precisely analyse the abundance and functional potential of Trp- and KYN-related enzyme genes, whilst combining fecal/serum targeted metabolomics to quantitatively detect key metabolites such as 5-HT, KYN, KYNA, and QUIN. Furthermore, resting-state functional magnetic resonance imaging (fMRI) and structural magnetic resonance imaging (MRI) techniques can be employed to comprehensively assess functional connectivity and changes in grey matter structure within brain regions associated with mood and cognition. Drawing on machine learning methods that classify neuroimaging subtypes based on indicators such as low-frequency amplitude, molecular features such as the “Trp metabolism abnormality subtype” and the “immune-inflammatory subtype” are integrated into imaging subtype classification, thereby enabling precise, individualized stratification based on the Trp-KYN metabolic axis (Hernández-Cacho et al., 2025; Li Z. et al., 2022; Tang L. et al., 2025).

On the other hand, the influence of gender, age, and comorbidities on the microbiota-Trp axis is a key issue requiring systematic stratified analysis in future research. Studies have shown that the GM in adulthood exhibits significant gender and age differences across the life cycle. Large-scale population studies have shown that the GM of pre-reproductive-age women exhibits greater diversity and contains a higher proportion of metabolically beneficial bacteria; these gender differences gradually diminish with advancing age (Zhang X. et al., 2021). Multi-omics studies in non-human primates and multi-species models have further revealed that differences between the proximal and distal colon in the KYN and 5-HT pathways widen with advancing age and the presence of intestinal inflammation; this suggests that gut location and age jointly influence the spatial distribution patterns of Trp metabolic pathways (Wang et al., 2024). These findings suggest that, in depression research, data from patients of different genders and age groups should not be simply pooled; rather, pre-stratification should be carried out at the study design stage, taking into account factors such as sex hormone levels, intestinal inflammation status, and metabolic indicators, in order to investigate the specific changes in the Trp-KYN pathway across different population subgroups.

With regard to comorbidity, patients with alcohol use disorder exhibit unique metabolic characteristics: during detoxification, their QUIN levels rise whilst KYNA levels fall, accompanied by changes in depressive symptoms and inflammatory markers. These changes are associated with specific SCFA-producing bacteria and their metabolites, indicating that substance use disorders and chronic inflammatory metabolic states can significantly influence the KYN metabolic profile (Leclercq et al., 2021). Furthermore, metabolome-microbiome association analyses in patients with MDD have revealed significant correlations between abnormalities in energy and lipid metabolism and specific GM and inflammatory factors (Amin et al., 2023; Lin et al., 2023). Multi-omics cognitive studies have further confirmed the existence of complex interactions between KYN pathway metabolites, cognitive dysfunction, and specific bacterial genera (Kennedy et al., 2017).

Consequently, future research on the microbiota-Trp-depression axis should explicitly stratify or group subjects according to inclusion criteria and statistical analysis: by gender and endocrine status, age group, and metabolic/inflammatory comorbidity to construct sub-cohorts, and conduct combined metagenomic, metabolomic, and neuroimaging modelling for each to analyse which subgroups are more likely to exhibit the “enhanced KYN toxicity-impaired neurotoxic brain network” pattern, and which present the “insufficient 5-HT synthesis-low emotional brain circuit activity” pattern. Building on this, future research could further correlate these stratified characteristics with clinical efficacy, thereby propelling research on Trp-microbiota-depression toward truly mechanism-driven precision psychiatry.

### 6.3 Prospects for novel therapeutic strategies

In addition to further exploring the application of traditional microbiome therapies, novel therapeutic strategies can be developed based on the mechanisms by which the microbiota regulates Trp metabolism. For example, synthetic biology techniques could be used to engineer classic probiotics or safe chassis bacteria to enable sustained, controllable production of Trp metabolites such as 5-HTP, indole, and their derivatives within the gut. This approach can circumvent the pharmacokinetic limitations associated with absorption and first-pass metabolism of orally administered small-molecule drugs. Recent research has shown that by introducing the plant-derived hydroxylase XcP4H into *Escherichia coli Nissle 1917*, followed by directed evolution and metabolic pathway optimisation, 5-HTP yield was successfully increased by a factor of 22. The resulting *EcN@5-HTP* strain increased gut and brain 5-HT levels and alleviated neuroinflammation and depression-like behaviors in mice, providing preclinical proof-of-concept for the use of engineered bacteria to modulate neuroactive Trp metabolites (Gao et al., 2025). Engineered bacteria also present biosafety challenges, including unintended persistence, environmental release, and horizontal gene transfer. Robust biocontainment and long-term safety evaluation are therefore required before clinical application. And this strategy remains at an early experimental stage, and its clinical translation will require rigorous evaluation of biosafety, genetic stability, dosing control, long-term colonization, and regulatory feasibility. This strategy can also be extended to the targeted synthesis of specific indole derivatives or modulators of the KYN pathway. For example, for metabolites such as IAA, IPA, and indole-3-carboxaldehyde (I3C), which have been shown to promote neurogenesis via AhR and possess anti-inflammatory effects, targeted production can be achieved by utilizing a chassis bacterium expressing specific Trp metabolic enzymes, thereby enabling precise control over ligand structure and dosage (Ghiboub et al., 2020; Wang X. et al., 2025).

Furthermore, as a key signaling node linking microorganisms, Trp metabolites, and immune and neural circuits, AhR has been shown in various stress and inflammation models to alleviate depression- and anxiety-like behaviors through activation by endogenous ligands. Studies have shown that various indole derivatives derived from microorganisms or the diet exert antidepressant-like effects in methamphetamine withdrawal-associated depression and anxiety via AhR, whilst AhR knockout completely abolishes this protective effect (Wang X. et al., 2025). Furthermore, gut-derived I3C can improve susceptibility to chronic stress, suppress neuroinflammation, and promote adult hippocampal neurogenesis by activating the AhR-SOCS2-NF-κB/NLRP3 pathway (Chen et al., 2025). Taken together, these findings suggest that through precise design of AhR ligands (including structure, affinity, and partial agonist/antagonist properties), as well as optimisation of administration sites and timing, it may be possible to exert anti-inflammatory, neuroplasticity-promoting, and vasoprotective effects whilst minimizing the potential toxicity associated with excessive AhR activation (Dong and Perdew, 2020; Ghiboub et al., 2020; Salminen, 2023). Future research could combine precisely designed AhR ligands with engineered bacterial strategies: one class of engineered bacteria would be responsible for sustained, low-dose production of beneficial indole ligands, whilst another class of engineered bacteria or exogenous drugs would act as local or short-acting AhR antagonists to suppress harmful, hyperactivated AhR pathways in specific tissues, thereby regulating the Trp-AhR signaling axis with higher spatio-temporal resolution (Mosa et al., 2024). In the long term, mapping an individual’s microbiota-Trp metabolism-AhR activity profile using multi-omics technologies, and individually matching engineered bacteria with AhR ligand libraries, is expected to enable precise intervention, i.e., determining which ligands a specific individual requires, in which intestinal segment, and with what release kinetics. This strategy will provide a novel therapeutic approach for depression and its comorbidities that combines both targeting and plasticity.

## 7 Conclusion

Current research on the “GM-Trp metabolism-depression” axis has established a relatively comprehensive chain of evidence across three complementary pathways: indole, 5-HT, and KYN. Regarding the KYN pathway, chronic stress models have shown that along the entire gut-brain axis from the colon to the hippocampus, there is upregulation of IDO1 and a significant increase in neurotoxic products such as KYN, 3-HK, and QUIN, which are closely associated with depression-like behavior. With regard to the 5-HT pathway, germ-free or microbiota-disrupted animals exhibit significant abnormalities in peripheral and central 5-HT levels; microbiota intervention can reshape intestinal 5-HT production and influence the vagal-central pathway, thereby regulating mood and pain processing. Research on the indole pathway highlights the “microbiota-indole-AhR-neuroimmunity” axis as a key hub: multiple studies indicate that indoles and their derivatives (IAA, IPA, I3C, etc.) derived from the microbiota can inhibit the NF-κB/NLRP3 inflammasome by activating AhR, thereby reducing inflammatory cytokine release and alleviating depression and anxiety. At the applied level, existing animal and preliminary clinical trials suggest that modulating the direction of Trp metabolism through specific bacterial strains, probiotics, or dietary strategies holds promise for personalized interventions for depression and its comorbidities. However, to truly translate this network into reproducible and scalable precision intervention strategies, there is an urgent need for in-depth interdisciplinary collaboration: psychiatry, neuroscience, microbiology, immunology, nutrition, and computational biology must be integrated within a single cohort, utilizing metagenomics, targeted metabolomics, and neuroimaging to track the multi-pathway remodeling of the microbiota-Trp-neuroimmune axis across different genders, ages, and comorbidities; simultaneously, multi-target intervention experiments targeting IDO/KYN pathway, AhR, and 5-HT signaling should be used to retrospectively validate the causal role of multi-pathway regulation on symptom dimensions and cognitive function. Through this cross-platform, interdisciplinary systemic integration, it is hoped that the currently relatively fragmented evidence chains regarding KYN, indole, and 5-HT can be consolidated into a “microbiota-Trp-neuroimmune” operational framework applicable to phenotypic diagnosis, treatment efficacy prediction, and targeted intervention design, thereby advancing the treatment of depression from empirical therapy toward mechanism-driven precision psychiatry.
